# Lithotomy;—Lecture and Operation

**Published:** 1861-10

**Authors:** 


### The Clinique.

### CHICAGO MEDICAL AND SURGICAL DISPENSARY.

Service, Prof. E. ANDREWS, Attending Surgeon.

Reported for the EXAMINER.

*Lithotomy.*—At my last clinical lecture, I sounded in your presence, a patient for calculus, and discovered a stone which I judged to be somewhat over an inch in diameter. To-day I shall bring the same patient before you, and perform the operation of lithotomy, or in solid Saxon words, "cutting for stone."

The first thing to be considered in relation to this operation, is the preparation of the patient. For this purpose you will first consider his general condition, particularly as to the presence of any aplastic diathesis. Experience shows that there is comparatively little danger of an acute plastic inflammation of fatal severity, hence, it will seldom be necessary to prepare the patient by reducing measures, such as purgatives, mercurials, and venesection; but on the other hand, there is great danger of an aplastic diathesis, which prevents the rapid gluing up and glazing over of the areolar interstices along the wound, and hence, allows a fatal infiltration of urine.

The most unmistakable sign marking the presence of this aplastic diathesis, is the aplastic inflammation resulting from any little pimples, scratches, or cuts which the patient may have. If any of these be present, and they show an unusual tendency to a weak suppuration, and an indisposition to heal readily, it is a bad sign, one which requires treatment before an operation is ventured on, for in such a diathesis the occurrence of urinary infiltration is greatly to be feared.

The best medicaments for the aplastic condition are the tonics. The patient should be fed liberally upon fresh meat and other substantial diet, and take per chloride of iron and quinine, freely, until the diathesis is thoroughly changed.

There are some neglected children in whom the presence of the stone keeps up incontinence of urine, by which their clothing is kept foul and their bodies made constantly to absorb ammoniacal and other effluviæ, thoroughly impairing the plasticity of the blood. In such cases it will be best to put the patient into a course of thorough cleanliness, where he will have the purest of fresh air, as well as tonics, for two months before an operation is ventured upon. In doubtful cases I would even test the patient's plasticity by making some slight incisions upon the thigh, and watching their appearance during the cure. It may be well to remark here, that it is not the reddest faces which mark the best constitution for operative purposes. The clear but solid white skin, such as is often seen in rheumatic patients, is a far better indication to a surgical eye, than the spongy, juicy redness of a toper's flesh.

Before the operation it is requisite to clear out the patient's rectum with an enema, otherwise the distention of that passage may bring its circumference in the way of the knife.

For the operation, you need four assistants, although you can get along in an emergency with two. One is to give the anæsthetic, one is to hold each thigh, and the fourth holds the staff. The latter should, if possible, be a surgeon, or at least, a very cool, intelligent layman.

The instruments required are, a catheter, a grooved staff, a sharp pointed scalpel, a straight bistoury, or a gorget, suitable forceps, and a tube to insert into the bladder, after the operatiou is over. You also want a syringe to wash out the bladder, and the usual supply of sponges, needles, ligatures, etc., to be used in case of hæmorrhage.

I will now direct the anæsthetic to be given, and as soon as the patient is under its influence, lay him upon this table, with his feet towards a strong light. We will now tie his hands securely to his ankles. This procedure can be dispensed with, but it is best to retain it, as at the critical moment of the incision into the bladder, any sudden motions of the patient might endanger his safety.

The hips are now drawn to the edge of the table, and I proceed to introduce a silver catheter into the bladder. The object of this is twofold. First, we wish to ascertain at the last moment that the stone is still there. This may strike you as a strange precaution, since we distinctly felt it and heard it click at our last sitting; but, gentlemen, I tell you some of the most distinguished surgeons in Europe have had the mortification of cutting into a bladder and finding no calculus, though they distinctly felt one at a previous examination. I can only account for this by supposing that the concretion had crumbled to pieces, and been discharged in the interval.

However this may be, surgeons are now cautious in the matter, and the rule is, that if we cannot feel the stone after the patient is actually on the table, we must defer the operation. Having introduced the catheter, I feel the same object as before, and as I percuss it, you distinctly hear the click, so the stone is doubtless still present. The second object of introducing the catheter is, that we may fill the bladder with warm water, and distend it so as to facilitate the operation of cutting into it. This being done, I will now introduce this grooved staff, and deliver it to the assistant to be held steady.

The staff must be held with the point well in the bladder, and the assistant must on no account, forget himself in that respect, for if he allow the point to draw back and escape from the canal after the urethra is opened, it is often nearly impossible to re-introduce it.

The next step is, to make the incision, which in this case we will do by what is called the lateral method. I first feel for the left *tuber ischii*, because along the inner border of this bone runs the internal pudic artery, which must not be cut. There are three main points to be avoided in the incision. One is the bulb of the urethra, or more accurately, the posterior part of the corpus spongiosum; the next is the rectum; and the third is the pudic artery. Between these three we have a sort of triangular space, within which an incision may be made down to the membranous portion of the urethra, without wounding any important organ. Placing, therefore, the point of my scalpel on the mesial line about an inch in front of the verge of the anus, I enter it well through the skin into the superficial fascia, and carry it backwards and outwards to the patient's left, towards a point between the anus and the *tuber ischii.* In this direction we shall avoid wounding both the latter organs. This first incision should be made pretty long, as the tissues of the perineum contract very much after cutting, owing to the presence of the dartoid tissue in portions of it.

The first incision being made, I will next deepen the anterior portion of it by dissecting carefully forwards and upwards toward the membranous portion of the urethra. At this stage of the operation, you will commonly cut the transversalis artery, which, if it does not cease its hæmorrhage by a little compression or torsion, may be tied. I am now able to feel the bend of the staff, and to distinguish the groove, into which I press the nail of the index finger. Now taking the scalpel again, I carry the point along the back of the finger, and follow the nail into the groove of the staff, cutting open the canal for about a third of an inch. Still keeping the finger there as a guide, the next step is to take a probe-pointed scalpel or straight bistoury, and placing the point of it in the groove, turn the edge to the left of the patient, and a little backwards, and push it into the bladder. The entrance will be known by the cessation of resistance, and by a gush of urine. In this step it is not desired to cut the prostate entirely through, lest you lay open the areolar spaces of the pelvis to urinary infiltration, but only to notch the gland freely, so as to allow the introduction of the finger. Withdrawing the bistoury, you insert the finger along the staff, using it as a wedge to enlarge the opening in the bladder, and feel for the calculus. Next, withdraw the staff and insert through the wound a pair of lithotomy forceps, carrying them along the finger as a guide. If you have contrived to retain hitherto most of the urine, it will be all the easier to seize the stone, which is the next step to be taken. By separating the blades freely, the calculus will generally roll between them and may be securely seized. After grasping it, there are two points to be attended to: first, that we do not grasp with it a fold of the bladder; and second, as calculi are often elongated, that it is not seized crosswise. That the bladder is not seized, may be ascertained by the finger, and by trying whether the forceps can be rotated on its axis easily. To ascertain that you have seized the stone endwise and not crosswise, feel the position of it by the finger, and if necessary, loosen the hold a little and rotate it into the proper position. If the calculus is a small one, it may be brought out in any position; if large, draw it gently and carefully down, much as you would extract a fœtus with obstetric forceps. Thus you see, I bring out the object which is oval in shape, and apparently an inch and a half in length.

The next step is to ascertain whether there are any more calculi present. With this view, I explore first with my finger, and next with an explorer or large steel sound. Finally, we will wash out the bladder by throwing in a copious stream of warm water with a syringe, to wash out any small fragments or clots of blood that may remain.

The great danger after this operation, is that of infiltration of urine into the areolar spaces of the pelvis. As a partial preventive, we will insert this silver tube through the wound into the bladder, and retain it there for thirty hours. The object is to carry off the urine in that way, until the effusion of plastic lymph has so glazed the surface of the wound as to render the infiltration impossible.

The mortality of this operation in European hospitals is very great; so much so, that one scarcely sees how it is justifiable to perform it there. In this western country, however, in private practice, the risk is much less, being, in a selection of favorable cases, perhaps one death in twenty, and one in ten or fifteen of all cases, taken promiscuously. There are many points of great interest connected with the pathology and treatment of this disease, which I have no time at present to detail, but must defer them to another opportunity.

---

NOTE.—This patient was under the care of Dr. H. Wanzer, originally, and attended by him after the operation. There were no untoward symptoms in the case. After five days the urine began to flow in its natural channel, and the boy recovered in the usual time.

